# Falsified Drugs in the Opinion of Patients Diagnosed with Cardiovascular Diseases—Nationwide and Cross-Sectional Study on the Example of EU-Member Country

**DOI:** 10.3390/ijerph18073823

**Published:** 2021-04-06

**Authors:** Damian Świeczkowski, Szymon Zdanowski, Piotr Merks, Miłosz Jaguszewski

**Affiliations:** 1 First Department of Cardiology, Faculty of Medicine, Medical University of Gdansk, Debinki 7, 80-211 Gdansk, Poland; damian.swieczkowski@gumed.edu.pl; 2 Department of Anaesthesiology and Intensive Therapy, Faculty of Medicine, Medical University of Gdansk, Mariana Smoluchowskiego 17, 80-214 Gdańsk, Poland; szymon.zdanowski@gumed.edu.pl; 3 Faculty of Medicine, Collegium Medicum, Cardinal Stefan Wyszynski University, 01-938 Warsaw, Poland; piotrmerks@googlemail.com; 4 Department of Pharmaceutical Technology, Faculty of Pharmacy, Collegium, Medicum in Bydgoszcz, 85-089 Bydgoszcz, Poland

**Keywords:** counterfeit medicine, falsified medicine, public health, Falsified Medicines Directive, European Union, pharmacist

## Abstract

Background: In light of a falsified medications pandemic, understanding the patient perspective on falsified medicines is warranted. Our study aimed to investigate the perspectives regarding falsified medicines among patients with cardiovascular diseases. Methods: Computer-assisted telephone interviews were conducted based on a questionnaire: (i) Respondents suffering from cardiovascular diseases and (ii) respondents not being chronically ill. Only participants below 50 years of age were included. Results: We enrolled 1200 respondents total, 800 in the study group and 400 in the control group (in cooperation with a professional public opinion research center). The vast majority of participants agreed that community pharmacies are the only place that ensures the secure purchasing of non-falsified drugs (67.01% study group and 65.25% control group; *p* < 0.01). The majority of respondents were convinced that purchasing medications on the Internet is associated with a higher risk of receiving falsified drugs. Patients diagnosed with cardiovascular diseases and those with “non-satisfactory financial situation” had significantly decreased likelihoods of obtaining a high score in general knowledge on falsified medications (OR = 0.64 and OR = 0.58, respectively). Conclusions: Awareness of the risks associated with falsified drugs among patients with cardiovascular diseases remains high but still insufficient.

## 1. Introduction

In 2005, The Centre for Medicines in the Public Interests estimated that falsified medicines’ global market might be worth roughly 70 billion dollars (58 billion Euros) by 2010 [1]. Recent data provided by the European Commission suggests that in 2015, customs officials seized about 40 million falsified drugs and estimated their value at 650 million Euros [2]. The Food and Drug Administration (FDA) has stated that between 2007–2008, 246 people died due to a serious allergic adverse event resulting from using falsified heparin [3]. It is worth mentioning that oncological drugs remain an important target of falsification, of which a good example is a bevacizumab (Avastin). In 2012, several batches of Avastin were falsified and did not contain any active pharmaceutical ingredients (API) [4]. According to the WHO, nearly 64% of antimalarial drugs dispensed in Africa should be considered falsified [5]. This problem has been observed not only in developing societies, but also in well-developed countries, which have at least, in theory, several protective mechanisms aimed at diminishing the dangers related to illegal drugs [6,7]. From a practical point of view, Poland, as a border country of the EU, remains a strategic place in preventing pharmaceutical crimes.

Consequently, the situation in Poland may have a direct impact on patient safety throughout Europe. In this context, it is worth recalling that in September 2016, near Bydgoszcz in central Poland, police discovered one of the first illegal pharmaceutical factories in Europe, and one of the largest in the world, and seized 430 thousand ampoules and tablets containing steroids and 100 thousand tablets for erectile dysfunction worth over 17 million PLN (Polish currency, approximately 4 million United States dollars) [8]. Finally, falsified medications should be perceived as a matter of vital importance, highlighting the need for interprofessional collaboration between agencies dedicated to health promotion, law enforcement authorities, as well as those responsible for creating drug policy, which can be seen via the example of cooperation between the WHO, the UN Office of Drugs and Crime (UNODC), and Interpol [9].

In light of this observation, we have identified in Europe intensified efforts to minimize the potential harm that the pandemic of falsified medications may cause to chronically ill patients, among others, those diagnosed with cardiovascular diseases. The new regulations, called the Falsified Medicines Directive (FMD), have recently been introduced in the European community. Thanks to these regulations, we can finally estimate the scale of the mentioned herein phenomenon throughout legal trade (particularly in community and hospital pharmacies). Secondly, it should ensure the safer profile of dispensed medications and increase access to more accurate data about routine drug dispensation [10]. It should be added that apart from new responsibilities for producers and wholesalers, one of the most critical issues which the FMD has introduced is authentication, defined as verification of the drug authenticity before dispensing it to the patient. In this sense, the community pharmacy will be dedicated to protecting the patient from falsified drugs from the patient’s perspective. Additionally, authentication will also be introduced into the hospital setting, influencing workflow and possibly generating additional costs and human efforts [11].

In light of the pandemic of falsified medications and recent legislative efforts, understanding the patient perspective on falsified medicines is warranted, both from the practical point of view and the impact on the current state of knowledge. Our study could be a starting point for designing educational initiatives to improve awareness of the risks related to falsified products among modern societies. The needs of patients suffering from cardiovascular diseases, particularly considering the epidemiology, should be regarded as especially important.

Our study aimed to investigate and understand the perspectives regarding falsified medications among patients diagnosed with cardiovascular diseases and then compare this perspective with patients not suffering from a chronic condition. The study also has educational value in that it contributes to improving patient knowledge about the phenomenon. Finally, our findings can also be understood in the context of public health and could be helpful for governments and other authorities responsible for drug policy in Europe.

## 2. Materials and Methods

### 2.1. Study Design and Sample Selection

This is an observational cross-sectional study using a quantitative approach in CATI’s applied form (Computer-assisted telephone interviewing). In studies based on CATI, interviews with respondents are conducted by phone, and the researcher or qualified pollster can use advanced software dedicated to collecting data. This process facilitates the collection of data and directly impacts the quality of the obtained information. The presented study was conducted simultaneously among two groups: (i) Respondents who declared being chronically ill and suffering from cardiovascular diseases (study group), and (ii) respondents who reported not being sick chronically (control). Only participants below 50 years of age were included in the mentioned above cohorts. Our research was conducted in cooperation with a professional public opinion research center (https://www.biostat.com.pl, accessed on 1 April 2021) and achieved fair representation in gender, age, and residence (geographical representative). No incentives were offered to participants.

### 2.2. Questionnaire Development—A Pilot Study, Face and Content Validity, Content

Our study is cross-sectional, in which the applied research tool was an authorial questionnaire prepared by the research team. This tool was evaluated during a pilot study among 110 respondents from a selected community pharmacy in Poland’s central part [12].

Due to financial and practical limitations (e.g., length of the questionnaire could be associated with the tiredness of respondents), after the pilot study, the questionnaire was shortened, and the final questions were selected after extensive discussion among the research team and an independent consultant employed in the pharmaceutical industry (face and content validity). The questionnaire was then also tested by the Gunning Fog index, achieving high and satisfactory text readability. Linguistic, stylistic, and grammatical correctness were assessed. Finally, the proper research tool was discussed with patients diagnosed with cardiovascular diseases (*n* = 10) and the patients’ opinions about the questionnaire were collected. In this final step of preparing the questionnaires, patients had the opportunity to share their opinion and provide tips/advice on how the tool could be improved.

The questionnaire consisted of seven questions and was divided into two parts. The first part (questions 1–3) provided information related to the diagnosis of cardiovascular diseases, hospital admissions within the past 12 months related to chest pain and/or myocardial infarction (MI), and the patient’s financial situation (the latter based on the previously met form of evaluation of the respondent’s economic condition used in the questionnaire and adapted to our needs). The second part (questions 4–7) aimed at a multidimensional evaluation of the patient’s perspective on falsified drugs, among others, in the context of understanding the definition of a falsified drug, epidemiological background, safety concerns, as well as the potential of falsification of the particular therapeutic group. Questions were based on a Likert scale. The questionnaire is attached in Supplementary File 1. The version attached in the appendix was translated into English by one of the research team members, an American Native Speaker with medical education and experience in proofreading academic articles.

### 2.3. Statistical Analysis

The statistical analysis was performed by an external company involved in collecting the data and experienced in data analysis for scientific purposes. Descriptive statistics and more advanced statistical methods were applied. Categorical variables are provided with percentages. We used the Pearson chi-square test to compare categorical variables. A two-sided P value of less than 0.05 was considered to indicate statistical significance.

For each of the questions listed in Table 1, a logistic regression model was constructed, and the odds ratios for all variables included in the model were presented. Patients were divided into groups based on the provided responses, i.e., for “1” patients with responses indicating a satisfactory level of knowledge (see Table 1); for “0” the others. The independent variables included sociodemographic features (age, gender, level of education), financial situation, and information about chronic diseases (coronary artery disease, heart failure, hypertension, diabetes). We decided to build our model based on the assumption that for each question, at least half of the responses should indicate a satisfactory level of knowledge (Table 1). Among the independent variables, we also included information regarding hospital admission due to chest pain or myocardial infarction during the previous 12 months. Our model used a backward stepwise approach, assuming that all independent variables were initially included, consequently eliminating those that contributed the least to the suggested model. The summary of regression analysis is attached in Supplementary File 2.

### 2.4. Ethical Consent

Our research was a non-interventional, cross-sectional survey conducted by an independent, external company (https://www.biostat.com.pl, accessed on 1 April 2021) In light of this, it was not necessary to obtain ethical consent to conduct the study. There was no risk to patients who participated in this study, and their participation in our research could prove to have some educational value. Interviews were collected outside of the clinical setting. Moreover, according to the Polish pharmaceutical law, ethical approval is not required for non-interventional studies [13].

## 3. Results

### 3.1. Setting

**Figure 1:**
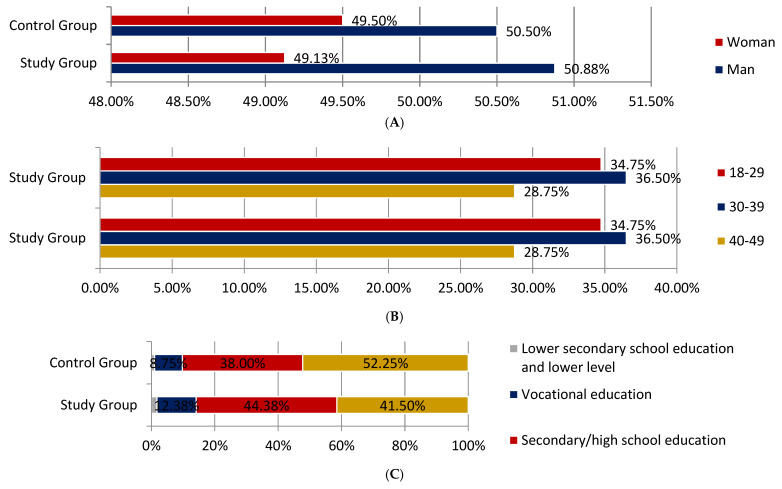
(**A**) Gender of respondents. (**B**) Age of respondents. (**C**) The level of education.

We enrolled a total of 1200 participants, 800 in the study group and 400 in control. More than 50% of respondents were men in both groups, while women constituted 49.13% and 49.50% surveyed (Figure 1A). Both in the control and the study group, individuals between 30 and 39 were the majority (36.5%) (Figure 1B). The study group was dominated by patients with secondary education, representing 44.38% of the total population. On the other hand, there were more respondents with higher education in the control group 52.25% (Figure 1C).

**Figure 2:**
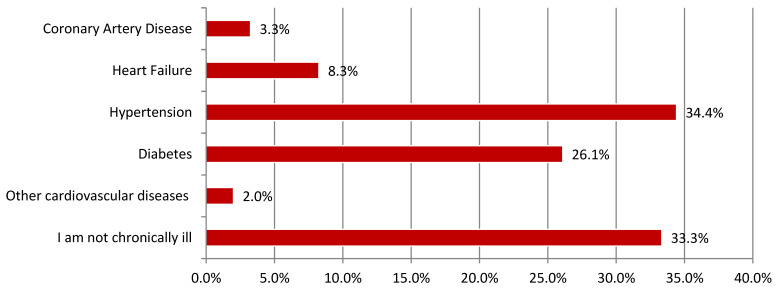
The chronic diseases among respondents (“I am not chronically ill” control group).

Arterial hypertension was the most commonly declared chronic disease. This answer was provided by almost one in every three individuals (34.42%). Coronary artery disease, heart failure, and other diseases affected fewer patients (3.25%, 8.25%, and 2.00%, respectively). A detailed distribution of responses has been summarized in Figure 2. The respondents who declared that they were not suffering from a chronic disease should be understood as the control group (33.33%).

**Figure 3:**
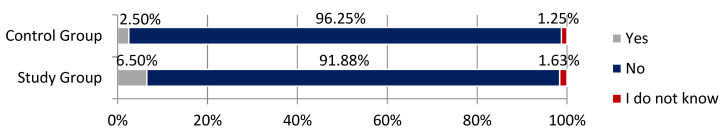
Hospital admission in the previous 12 months due to chest pain or myocardial infarction (MI).

During a review of the patient’s health over the past 12 months, admission to a hospital due to the occurrence of chest pain or/and myocardial infarction was required only in a small number of our participants. Hospitalization occurred in 6.5% of cases in the study group and 2.5% in the control group (Figure 3).

**Figure 4:**
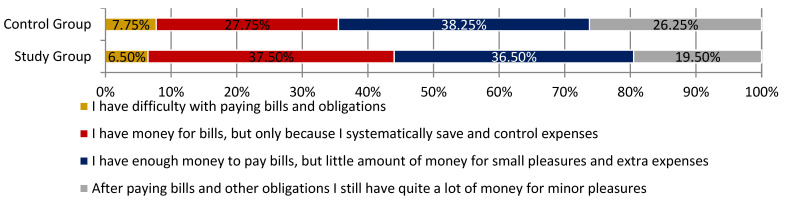
The financial status of respondents.

Respondents were then requested to assess their financial status. Participants in the study cohort frequently claimed that they have enough financial sources (money) to pay the bills (accounts). However, they believe this is only due to regularly saving money and controlling their expenses (37.50%) (Figure 4).

### 3.2. Main Findings

In both the study and the control groups, participants mostly agreed with the statement that a falsified drug does not contain any active pharmaceutical ingredient or contains the incorrect amount of API (respectively, 74.63% and 71.50%). Both groups replied similarly to the statement that falsified drugs have poisonous substances, with less than half of the study group’s respondents, and those in control assessing this statement as pertinent (respectively, 43.63% and 41.00% of respondents). According to an overwhelming majority of respondents, both in the study and the control group, falsified medicines contain the wrong amount of API, which is correct (76.88% and 79.00%, respectively).

**Figure 5:**
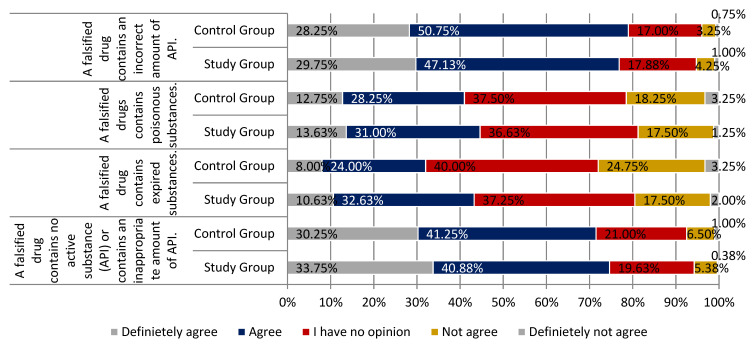
Falsified drugs in the opinion of respondents from the study and control group part 1.

**Figure 6:**
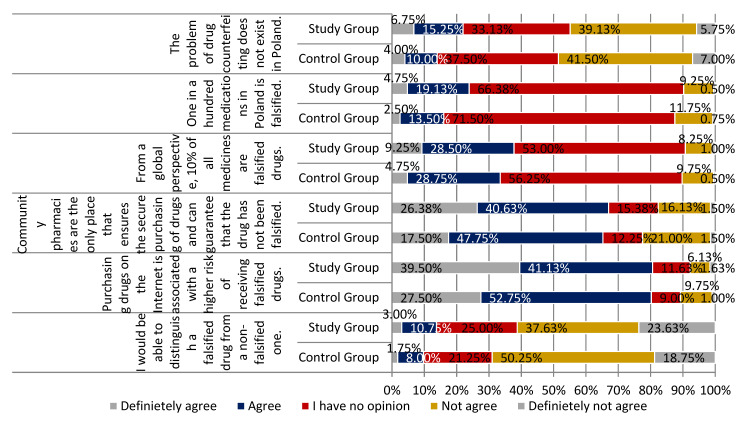
Falsified drugs in the opinion of respondents from study and control group part 2.

**Figure 7:**
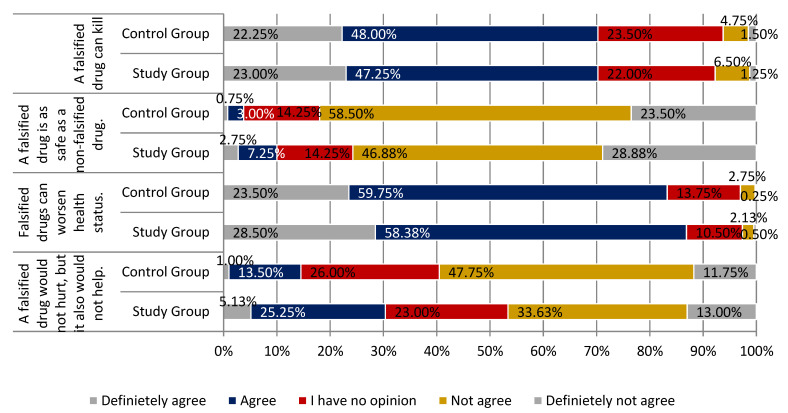
Falsified drugs in the opinion of respondents from the study and control group part 3.

Few individuals agreed with the statement that the problem of drug counterfeiting does not exist in Poland, with a slightly higher percentage of positive answers identified among respondents from the study group (22.00% vs. 14.00% from the control group). The difference in the distributions of responses in each group was statistically significant (*p* < 0.05). The vast majority of participants agreed that community pharmacies are the only place that ensures the secure purchasing of drugs and guarantees that the drug has not been falsified (67.01% study group and 65.25% control group; *p* < 0.01). The majority of respondents were convinced that purchasing drugs on the Internet is associated with a higher risk of receiving falsified drugs. More than 80% of the respondents agreed with this statement 80.63% of respondents from the study group and 80.25% of the control group respondents (definitely yes, somewhat yes). The difference in the distributions of responses in each group was statistically significant (*p* < 0.001). More than half of the participants claimed that they could not distinguish a falsified drug from a non-falsified one. There were slightly more respondents who felt this way in the control group than in the study group (69.00% and 61.26%; *p* < 0.01). Participants agreed that falsified drugs can worsen the health status (86.88% of respondents in the study group and 83.25% in the control group). The vast majority of respondents did not agree with the statement that a falsified drug is as safe as a non-falsified drug (study group 75.76% and control group 82.30%; *p* < 0.001). All the above-mentioned main findings are summarized in Figure 5, Figure 6 and Figure 7.

**Figure 8:**
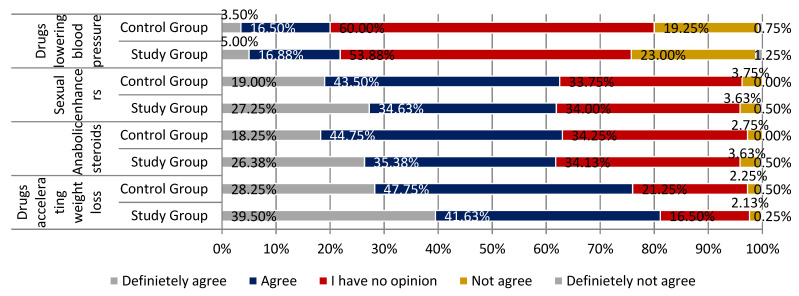
The most frequently falsified medicines in the opinion of respondents summary (part 1).

**Figure 9:**
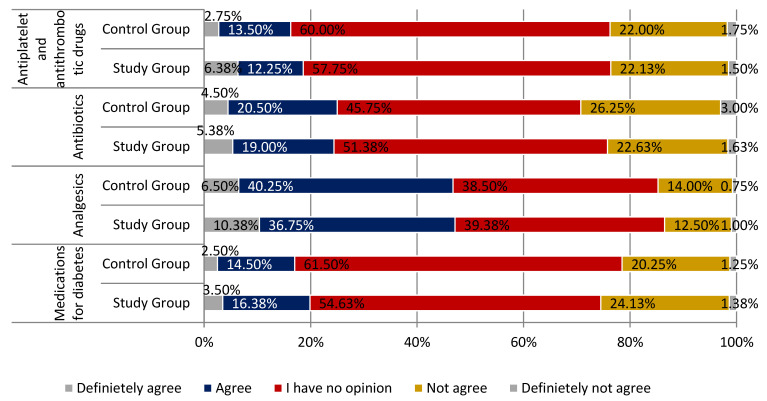
The most frequently falsified medicines in the opinion of respondents summary (part 2).

Respondents from both groups agreed with the statement that the most commonly falsified drugs are those accelerating weight loss. A larger percentage of people convinced this statement is true was observed in the study group (respectively, 81.13% of respondents in the study group and 76.00% in the control group; *p* < 0.01). Compared to the previous statement, a slightly smaller number of respondents considered anabolic steroids as the most common drugs that are falsified (respectively, 61.76% and 63.00% of responses; *p* < 0.01). In the case of drug potency, the distribution of responses was similar to that achieved in the context of anabolic steroids. Sexual enhancers were believed to be the most commonly falsified drugs. A small group of respondents agreed with the statement that antiplatelet and antithrombotic drugs are the most commonly falsified drugs (18.63% of respondents from the study group and 16.25% of the respondents from the control group). Every fourth respondent did not consider this sentence correct (23.63% and 23.75% of respondents). This part of the questionnaire is summarized in Figure 8 and Figure 9 (parts 1 and 2).

### 3.3. Logistic Regression

The results obtained from logistic regression indicated that individuals who had a higher education degree had 70% higher odds of selecting an answer, indicating a satisfactory level of knowledge in question four (Table 1). For patients diagnosed with diabetes, these odds increased by 40%. The result in question five was significantly influenced by whether the patient suffered from chronic cardiovascular disease and was hospitalized during the last 12 months (*p* < 0.05). Patients diagnosed with cardiovascular diseases had lower odds of obtaining a score indicating high knowledge, i.e., three or more responses indicating a satisfactory level of knowledge (OR = 0.54). However, those hospitalized patients were almost twice as likely to get a high score as the rest of the population (OR = 1.89). In this model, only the ratio with the variable’ non-satisfactory financial situation’ was significantly different from zero (*p* < 0.001). The chance to get a high score in question six was 71% lower in this group compared with the rest of the population.

In summary, patients diagnosed with cardiovascular diseases and those with a “non-satisfactory financial situation” had significantly decreased likelihoods of obtaining a high score in general knowledge on falsified medications, based on the number of responses indicating the satisfactory level of knowledge. Both variables decreased this chance by 40% (respectively, OR = 0.64 and OR = 0.58). A detailed summary of the logistic regression model is described in tables (Table 2, Table 3, Table 4, Table 5 and Table 6) and Supplementary File 2.

## 4. Discussion

The presented study is the first example in the world of representative research conducted among participants with self-declared cardiovascular diseases aimed at defining the patients’ perspective on falsified (counterfeited) medicinal products. Implementation of a specific protocol based on the study (patients with self-declared cardiovascular diseases) and the control group (not chronically ill) should be considered a unique motive in social pharmacy and the social medicine disciplines. Recently, falsified drugs have become an essential topic in cardiology. Antignac et al. [14] evaluated that amlodipine and captopril were the most frequently identified cardiovascular drugs with poor quality in Africa.

It should be noted that the results obtained from research conducted thus far have led to highly contradictory and incomplete information. This could be due to the lack of fair representation in prior research groups. In previous studies, one can find a juxtaposition of the healthcare professionals’ perspective compared to the layperson’s perspective. To the best of our knowledge, our study is the first scientific attempt to confront opinions on falsified medications among the chronically ill versus those not chronically ill [15,16]. In light of these observations, our research not only helps bridge the gap in current knowledge and remains attractive from a theoretical point of view, but also provides a strong practical background.

Respondents defined falsified medications as a product not containing an active pharmaceutical ingredient or an incorrect API quantity. Participants were sure about the higher risk associated with purchasing drugs on the Internet than in more traditional ways; however, respondents from the study group (self-declared occurrence of cardiovascular diseases) were more convinced of this. Indeed, online purchasing remains an important place for the distribution of falsified drugs [17,18]. These results are optimistic because most participants were aware of the existing dangers related to a non-traditional way of delivering medicinal products. These findings are essential in light of recent studies highlighting the almost unlimited accessibility of anabolic steroids and testosterone via the Internet. Such products are characterized by their very high potential of falsification [19]. Purchasing medicinal products via the Internet remains the main reason for patients having drugs containing falsified API or not containing any active substances. Such a scenario was seen in Japan with anti-obesity products, where scientists identified sibutramine in the herbal supplements [20]. In our study, respondents agreed that community pharmacy subsets were the only place that guaranteed the safety and quality of drugs (the availability of non-falsified medicines). Respondents from the study group were more convinced of this statement. The participants were convinced that they would not distinguish falsified medications and non-falsified drugs, with the respondents from the study group slightly more confident in this matter. Following the patients’ perspectives, the most frequently falsified medications are believed to be drugs included in the following groups: Anti-obesity, anabolic steroids, those for erectile dysfunction, and more rarely, analgesics. More respondents in the study group supported the appropriateness of this statement. Many participants did not have a sufficient level of knowledge to assess the possibility of falsification among hypertensives, antidiabetics, antibiotics, antiplatelet, or antithrombotic agents.

This research can support the designing and implementation of interventions or educational programs dedicated to improving patients’ awareness in the context of the potential harm of falsified medicines. Thus far, the actions provided by governments and international organizations have been focused on chain distribution or implementing new initiatives, e.g., Falsified Medicines Directive (FMD) by the European Commission [21,22]. Undoubtedly, authentication seems to be a reasonable solution to detect falsified, expired, or recalled drugs, even if we anticipate that some technical problems may occur during this process [23,24]. Experts have suggested that authentication should be supported by an advanced tool such as software with audio and visual feedback, which can improve the accuracy of detection and positively impact the pharmacist’s awareness [25]. Educational programs and a more systematic approach to the problem mentioned can minimize the potential harm for the patient and raise the awareness of falsification in the public domain [26]. The introduction of FMD is also crucial from a pharmacovigilance point of view, leading to an easier gathering of adverse events and signal monitoring [27]. To adequately understand our findings, it is worth mentioning that not only in Europe or in the United States have governments intensified their efforts to combat the pandemic of falsified drugs, but more strict rules have also been introduced to the Middle East, e.g., in the Iranian legal framework [28], or into Canadian law [29].

Our study has several limitations. The data obtained during this research is based on the respondents’ declarations and were not confronted with more objective, clinical information. Moreover, we are familiar with the fact that various terms describe fake drugs, falsified or counterfeit medications. Apart from legal matters, these terms can be used interchangeably. Recently, a new term has been coined, which is to be understood as a compromise “falsified (counterfeit) medicines (FFCms)” [30].

## 5. Conclusions

Awareness of the risks associated with falsified drugs among respondents is high, higher still among patients with cardiovascular diseases, but remains insufficient in both groups. Many respondents did not have a sufficient level of knowledge to assess the possibility of falsification among hypertensives, antidiabetics, antibiotics, antiplatelets, or antithrombotic agents. The presented results should be understood as an important voice in the global discussion about the falsification of medications as a challenge for public health. Our results also facilitate the design of interventions and educational programs to increase the awareness of modern society regarding the pandemic of falsified medications.

## Figures and Tables

**Table 1:** Responses indicating a satisfactory level of knowledge.

	Responses Indicating the Satisfactory Level of Knowledge
Question Four	
A falsified drug contains no active substance (API) or has an inappropriate amount of API.	definitely agree, agree
A falsified drug contains expired substances.	definitely agree, agree
A falsified drugs contains poisonous substances.	definitely agree, agree
A falsified drug contains an incorrect amount of API.	definitely agree, agree
Question Five	
The problem of drug counterfeiting does not exist in Poland.	I do not agree, definitely do not agree
One in a hundred medications in Poland is falsified.	definitely agree, agree
From a global perspective, 10% of all medicines are falsified drugs.	definitely agree, agree
Community pharmacies are the only place that ensures the secure purchasing of drugs and can guarantee that the drug has not been falsified.	definitely agree, agree
Purchasing drugs on the Internet is associated with a higher risk of receiving falsified drugs.	definitely agree, agree
I would be able to distinguish a falsified drug from a non-falsified one.	I do not agree, definitely do not agree
Question Six	
A falsified drug would not hurt, but it also would not help.	I do not agree, definitely do not agree
Falsified drugs can worsen the health status.	definitely agree, agree
A falsified drug is as safe as a non-falsified drug.	I do not agree, definitely do not agree
A falsified drug can kill.	definitely agree, agree
Question Seven	
Drugs accelerating weight loss	definitely agree, agree
Anabolic steroids	definitely agree, agree
Sexual enhancers	definitely agree, agree
Drugs lowering blood pressure	definitely agree, agree
Medications for diabetes	definitely agree, agree
Analgesics	definitely agree, agree
Antibiotics	definitely agree, agree
Antiplatelet and antithrombotic drugs	definitely agree, agree

**Table 2:** Question four—logistic regression model; two or more responses indicating the satisfactory level of knowledge.

	Estimator	Standard Error	z	*p*-Value
Secondary/high school education	0.01	0.21	0.03	0.97
Higher education	0.53	0.21	2.5	0.01
Diabetes	0.34	0.17	2	0.04
Intercept	0.92	0.19	4.9	0

**Table 3:** Question five—logistic regression model; three or more responses indicating the satisfactory level of knowledge.

	Estimator	Standard Error	z	*p*-Value
Cardiovascular diseases	−0.62	0.19	−3.2	0
Hospitalization	0.64	0.33	1.9	0.05
Intercept	0.92	0.07	13	0

**Table 4:** Question six—logistic regression model; two or more responses indicating the satisfactory level of knowledge.

	Estimator	Standard Error	z	*p*-Value
Hypertension	0.31	0.19	1.7	0.09
Hospitalization	−0.54	0.33	−1.6	0.1
Financial situation—good	−0.03	0.24	−0.14	0.89
Financial situation—satisfactory	−0.19	0.24	−0.79	0.43
Financial situation—non-satisfactory	−1.2	0.31	−4	0
Intercept	2	0.2	9.8	0

**Table 5:** Question seven—logistic regression model; four or more responses indicating the satisfactory level of knowledge.

	Estimator	Standard Error	z	*p*-Value
Gender—Male	0.19	0.12	1.6	0.1
Diabetes	0.22	0.13	1.7	0.09
Hospitalization	0.41	0.26	1.5	0.12
Intercept	−0.45	0.09	−4.9	0

**Table 6:** All questions—logistic regression model; eleven or more responses indicating the satisfactory level of knowledge.

	Estimator	Standard Error	z	*p*-Value
Age 30–39	0.27	0.15	1.9	0.06
Age 40–49	0.01	0.15	0.08	0.93
Cardiovascular diseases	−0.45	0.19	−2.3	0.02
Hospitalization	0.49	0.3	1.6	0.1
Financial situation—good	0.08	0.17	0.48	0.63
Financial situation—satisfactory	−0.19	0.17	−1.1	0.26
Financial situation—non-satisfactory	−0.54	0.26	−2.1	0.04
Intercept	0.68	0.16	4.2	0

## Data Availability

Not applicable.

## Supplementary Materials

The following are available online at https://www.mdpi.com/article/10.3390/ijerph18073823/s1, Supplementary File 1 Falsified drugs in the opinion of patients diagnosed with cardiovascular diseases, and Supplementary File 2 RESULTS—LOGISTIC REGRESSION MODEL—detailed information.

## References

[1] Lee K.S., Yee S.M., Zaidi S.T.R., Patel R.P., Yang Q., Al-Worafi Y.M., Ming L.C. (2017). Combating Sale of Counterfeit and Falsified Medicines Online: A Losing Battle. Front. Pharmacol..

[2] Coraz Więcej Fałszywych Leków Produkowanych Jest w Polsce—PRAWO. https://www.rynekaptek.pl/prawo/coraz-wiecej-falszywych-lekow-produkowanych-jest-w-polsce,17016.html.

[3] Lawsuit: Heparin Caused Dialysis Patient’s Death Breckinridge County Man Died a Day After Receiving Blood Thinner—Nolan Law Group. http://nolan-law.com/lawsuit-heparin-caused-dialysis-patient’s-death/.

[4] Mackey T.K., Cuomo R., Guerra C., Liang B.A. (2015). After counterfeit Avastin^®^—What have we learned and what can be done?. Nat. Rev. Clin. Oncol..

[5] Fake Pharmaceuticals—Bad Medicine|International|The Economist. https://www.economist.com/international/2012/10/13/bad-medicine.

[6] Fayzrakhmanov N. (2015). Fighting trafficking of falsified and substandard medicinal products in Russia. Int. J. Risk Saf. Med..

[7] Suleman S., Woliyi A., Woldemichael K., Tushune K., Duchateau L., DeGroote A., Vancauwenberghe R., Bracke N., De Spiegeleer B. (2016). Pharmaceutical Regulatory Framework in Ethiopia: A Critical Evaluation of Its Legal Basis and Implementation. Ethiop. J. Health Sci..

[8] Zlikwidowano Fabrykę Nielegalnie Produkowanych Leków—PRAWO. https://www.rynekaptek.pl/prawo/zlikwidowano-fabryke-nielegalnie-produkowanych-lekow,16088.html.

[9] Mackey T.K., Liang B. (2013). Improving global health governance to combat counterfeit medicines: A proposal for a UNODC-WHO-Interpol trilateral mechanism. BMC Med..

[10] Merks P., Swieczkowski D., Byliniak M., Drozd M., Krupa K., Jaguszewski M., Brindley D., Naughton B.D. (2016). The European Falsified Medicines Directive in Poland: Background, implementation and potential recommendations for pharmacists. Eur. J. Hosp. Pharm..

[11] Smith G., Smith J., Brindley D. (2014). The Falsified Medicines Directive: How to secure your supply chain. J. Generic Med. Bus. J. Generic Med. Sect..

[12] [2017/Nr 2] Leki Sfałszowane w Opinii Pacjentów Polskich Aptek—Jednoośrodkowe Badanie. Farmacja Polska—PTFarm. https://ptfarm.pl/wydawnictwa/czasopisma/farmacja-polska/103/-/26942.

[13] (2008). Pharmaceutical Law Act of 6 September 2001. J. Laws.

[14] Antignac M., Diop B.I., De Terline D.M., Bernard M., Do B., Ikama S.M., N’Guetta R., Balde D.M., Tchabi Y., Aly A.S. (2017). Fighting fake medicines: First quality evaluation of cardiac drugs in Africa. Int. J. Cardiol..

[15] Binkowska-Bury M., Januszewicz P., Wolan M., Sobolewski M., Krauze M., Fijalek Z. (2012). Counterfeit medicines in Poland: Opinions of primary healthcare physicians, nurses and lay persons. J. Clin. Nurs..

[16] Binkowska-Bury M., Wolan M., Januszewicz P., Mazur A., Fijalek Z.E. (2012). What Polish Hospital Healthcare Workers and Lay Persons Know about Counterfeit Medicine Products?. Cent. Eur. J. Public Health.

[17] Sanada T., Yoshida N., Matsushita R., Kimura K., Tsuboi H. (2020). Falsified Tadalafil Tablets Distributed in Japan via the Internet. Forensic Sci. Int..

[18] Gaudiano M.C., Borioni A., Antoniella E., Valvo L. (2016). Counterfeit Adderall Containing Aceclofenac from Internet Pharmacies. J. Forensic Sci..

[19] McBride J.A., Carson C.C., Coward R.M. (2016). The Availability and Acquisition of Illicit Anabolic Androgenic Steroids and Testosterone Preparations on the Internet. Am. J. Men’s Health.

[20] Yoshida N., Numano M., Nagasaka Y., Ueda K., Tsuboi H., Tanimoto T., Kimura K. (2015). Study on health hazards through medicines purchased on the Internet: A cross-sectional investigation of the quality of anti-obesity medicines containing crude drugs as active ingredients. BMC Complement. Altern. Med..

[21] Hamilton W.L., Doyle C., Halliwell-Ewen M., Lambert G. (2016). Public health interventions to protect against falsified medicines: A systematic review of international, national and local policies. Health Policy Plan..

[22] Naughton B.D., Smith J., Brindley D. (2015). Establishing good authentication practice (GAP) in secondary care to protect against falsified medicines and improve patient safety. Eur. J. Hosp. Pharm..

[23] Naughton B., Roberts L., Dopson S., Chapman S., Brindley D. (2016). Effectiveness of medicines authentication technology to detect counterfeit, recalled and expired medicines: A two-stage quantitative secondary care study. BMJ Open.

[24] Mackey T.K., Nayyar G. (2017). A review of existing and emerging digital technologies to combat the global trade in fake medicines. Expert Opin. Drug Saf..

[25] Naughton B., Roberts L., Dopson S., Brindley D., Chapman S. (2017). Medicine authentication technology as a counterfeit medicine-detection tool: A Delphi method study to establish expert opinion on manual medicine authentication technology in secondary care. BMJ Open.

[26] Ziance R.J. (2008). Roles for pharmacy in combatting counterfeit drugs. J. Am. Pharm. Assoc..

[27] Beninger P. (2017). Opportunities for Collaboration at the Interface of Pharmacovigilance and Manufacturing. Clin. Ther..

[28] Kebriaeezadeh A., Zaboli P., Hashemi-Meshkini A., Varmaghani M., Gholami H., Vazirian I., Zekri H.-S., Eslamitabar S. (2016). Pharmaceutical laws and regulations in Iran: An overview. J. Res. Pharm. Pract..

[29] Attaran A. (2015). Stopping Murder by Medicine: Introducing the Model Law on Medicine Crime. Am. J. Trop. Med. Hyg..

[30] Anđelković M., Björnsson E., De Bono V., Dikić N., Devue K., Ferlin D., Hanževački M., Jónsdóttir F., Shakaryan M., Walser S. (2017). The development and appraisal of a tool designed to find patients harmed by falsely labelled, falsified (counterfeit) medicines. BMC Health Serv. Res..

