# Supplementary material for: Falsified Drugs in the Opinion of Patients Diagnosed with Cardiovascular Diseases—Nationwide and Cross-Sectional Study on the Example of EU-Member Country
Source: Int J Environ Res Public Health. 2021 Apr 6;18(7):3823. doi: 10.3390/ijerph18073823 (PMC8038753; doi:10.3390/ijerph18073823)
Supplement: Supplementary file 1 [file ijerph-18-03823-s001.zip › Supplementary_file_1.docx]

**Falsified drugs in the opinion of patients diagnosed with cardiovascular diseases**

**Supervisor of the project: dr. hab. n. med. Miłosz Jaguszewski, FESC**

**STUDY POPULATION**

**(inclusion criteria under 50 years of age,**

**representative in regards to gender, age and place of residence)**

400 respondents

800 respondents

Study group:

Respondents who declared being chronically ill, and suffering from cardiovascular diseases.

(answer: Yes, question 1)

Control Group:

Respondents who declared not being chronically ill

(answer: No, question 1)

**1. Are you chronically ill? (MORE than 1 response can be selected)**

 Yes – coronary artery disease

 Yes – heart failure

 Yes - hypertension

 Yes – diabetes mellitus

 Yes – other cardiovascular chronic disease, (which? .....)

 Not chronically ill

 Not applicable (the respondent did not select any of the answers above, and IS NOT in the control group nor the study group).

**2. Were you hospitalized in the past 12 months due to chest pain or a myocardial infarction?**

 Yes

 No

 I don't remember

 I don't understand the question

**3. Please select the statement, which best describes your actual financial situation?**

 After paying my bills and other responsibilities, I still have a lot of money left for spending

 I have a lot of money to pay my bills, but only a little bit of money for small pleasures and additional expenses

 I have enough money to pay my bills, but only because I systematically save money and control my expenses
 I have trouble paying my bills and other responsibilities.

# 4. Please respond to the following statements:

|  | Definitely agree | I agree | No opinion | I do not agree | Definitely do not agree |
| --- | --- | --- | --- | --- | --- |
| A falsified drug contains no active substance (API) or contains an inappropriate amount of API. | 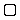 | 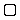 | 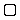 | 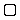 | 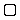 |
| A falsified drug contains expired substances. | 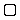 | 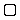 | 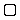 | 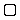 | 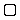 |
| A falsified drugs contains poisonous substances. | 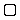 | 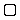 | 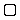 | 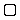 | 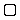 |
| A falsified drug contains an incorrect amount of API. | 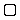 | 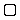 | 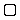 | 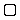 | 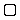 |

**5. Please respond to the following statements:**

|  | Definitely agree | I agree | | No opinion | I do not agree | Definitely do not agree |
| --- | --- | --- | --- | --- | --- | --- |
| The problem of drug counterfeiting does not exist in Poland. | 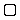 | 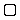 | | 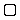 | 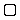 | 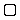 |
| One in a hundred medications in Poland is falsified. | 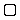 | 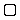 | | 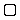 | 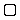 | 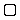 |
| From a global perspective, 10% of all medicines are falsified drugs. | 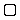 | 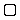 | | 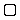 | 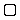 | 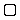 |
| Community pharmacies are the only place that ensures the secure purchasing of drugs and can guarantee that the drug has not been falsified. | 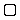 | 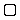 | | 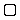 | 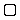 | 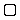 |
| Purchasing drugs on the Internet is associated with a higher risk of receiving falsified drugs. | 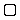 | | 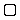 | 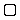 | 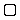 | 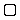 |
| I would be able to distinguish a falsified drug from a non-falsified one. | 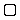 | 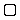 | | 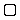 | 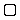 | 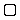 |

**6. Please respond to the following statements regarding the use of falsified medications:**

|  | Definitely agree | I agree | No opinion | I do not agree | Definitely do not agree |
| --- | --- | --- | --- | --- | --- |
| A falsified drug would not hurt, but it also would not help. | 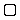 | 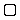 | 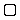 | 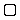 | 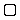 |
| Falsified drugs can worsen the health status. | 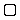 | 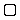 | 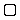 | 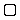 | 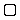 |
| A falsified drug is as safe as a non-falsified drug. | 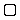 | 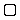 | 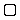 | 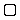 | 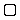 |
| A falsified drug can kill. | 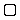 | 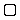 | 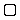 | 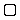 | 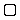 |

**7. Do you agree that the most frequently falsified drugs are:**

|  | Definitely agree | I agree | No opinion | I do not agree | Definitely do not agree |
| --- | --- | --- | --- | --- | --- |
| Drugs accelerating weight loss | 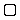 | 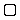 | 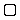 | 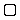 | 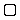 |
| Anabolic steroids | 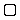 | 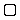 | 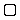 | 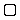 | 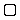 |
| Sexual enhancers | 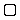 | 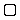 | 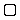 | 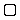 | 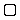 |
| Drugs lowering blood pressure | 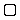 | 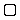 | 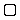 | 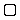 | 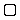 |
| Medications for diabetes | 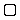 | 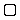 | 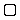 | 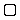 | 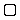 |
| Analgesics | 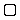 | 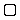 | 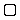 | 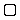 | 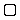 |
| Antibiotics |  |  |  |  |  |
| Antiplatelet and antithrombotic drugs |  |  |  |  |  |
