# Supplementary material for: Falsified Drugs in the Opinion of Patients Diagnosed with Cardiovascular Diseases—Nationwide and Cross-Sectional Study on the Example of EU-Member Country
Source: Int J Environ Res Public Health. 2021 Apr 6;18(7):3823. doi: 10.3390/ijerph18073823 (PMC8038753; doi:10.3390/ijerph18073823)
Supplement: Supplementary file 1 [file ijerph-18-03823-s001.zip › Supplementary_file_2.docx]

****RESULTS – LOGISTIC REGRESSION MODEL – detailed information****

**Falsified drugs in the opinion of patients diagnosed with cardiovascular diseases – nationwide and cross-sectional study on the example of EU-member country**

***

Based on the information derived and cited in the article we summarized patient knowledge on counterfeit drugs. This distribution is the basis of logistic regression model.

**Financial situation:**

GOOD - After paying my bills and other responsibilities, I still have a lot of money left for spending

SATISFACTORY - I have a lot of money to pay my bills, but only a little bit of money for small pleasures and additional expenses; I have enough money to pay my bills, but only because I systematically save money and control my expenses

NON-SATISFACTORY - I have trouble paying my bills and other responsibilities.

***

**Table 1. A falsified drug contains no active substance (API) or contains an inappropriate amount of API.**

|  | Estimator | Standard error | z | p-value |
| --- | --- | --- | --- | --- |
| Secondary/high school education | 0.1062 | 0.2027 | 0.524 | 0.6003 |
| Higher education | 0.3939 | 0.2055 | 1.917 | 0.05525 |
| Diabetes | 0.2632 | 0.155 | 1.698 | 0.08957 |
| Financial situation – good | -0.1043 | 0.184 | -0.5667 | 0.5709 |
| Financial situation – satisfactory | -0.2014 | 0.1857 | -1.084 | 0.2783 |
| Financial situation – non- satisfactory | -0.7455 | 0.2714 | -2.747 | 0.006016 |
| Intercept | 0.9091 | 0.2298 | 3.956 | 7.606e-05 |

*******

**Table 2. A falsified drug contains expired substances.**

|  | Estimator | Standard error | Z | p-value |
| --- | --- | --- | --- | --- |
| Hypertension | 0.3657 | 0.1235 | 2.961 | 0.003069 |
| Intercept | -0.5551 | 0.07406 | -7.496 | 6.579e-14 |

*******

**Table 3. A falsified drugs contains poisonous substances.**

|  | Estimator | Standard error | z | p-value |
| --- | --- | --- | --- | --- |
| Secondary/high school education | -0.4377 | 0.1863 | -2.349 | 0.01882 |
| Higher education | -0.2847 | 0.1845 | -1.543 | 0.1228 |
| Hypertension | 0.2937 | 0.1261 | 2.328 | 0.01989 |
| Diabetes | 0.2056 | 0.1365 | 1.506 | 0.132 |
| Intercept | -0.1086 | 0.1749 | -0.6212 | 0.5345 |

*******

**Table 4. A falsified drug contains an incorrect amount of API.**

|  | Estimator | Standard error | z | p-value |
| --- | --- | --- | --- | --- |
| Age 30-39 | 0,3409 | 0.1676 | 2.034 | 0.04196 |
| Age 40-49 | 0.06243 | 0.1717 | 0.3636 | 0.7162 |
| Secondary/high school education | -0.00173 | 0.214 | -0.008085 | 0.9935 |
| Higher education | 0.378 | 0.2197 | 1.72 | 0.0854 |
| Intercept | 0.9468 | 0.2151 | 4.401 | 1.077e-05 |

***

**Table 5. The problem of drug counterfeiting does not exist in Poland.**

|  | Estimator | Standard error | z | p-value |
| --- | --- | --- | --- | --- |
| Secondary/high school education | -0.3738 | 0.1888 | -1.98 | 0.04766 |
| Higher education | -0.188 | 0.1881 | -0.9992 | 0.3177 |
| Cardiovascular diseases | -0.5586 | 0.1951 | -2.863 | 0.004193 |
| Financial situation – good | -0.2269 | 0.1571 | -1.445 | 0.1486 |
| Financial situation – satisfactory | -0.4435 | 0.1612 | -2.752 | 0.005927 |
| Financial situation – non- satisfactory | -0.5569 | 0.2599 | -2.143 | 0.03214 |
| Intercept | 0.4177 | 0.2069 | 2.019 | 0.0435 |

*******

**Table 6. One in a hundred medications in Poland is falsified.**

|  | Estimator | Standard error | z | p-value |
| --- | --- | --- | --- | --- |
| Hypertension | 0.2854 | 0.1516 | 1.883 | 0.05968 |
| Diabetes | 0.3146 | 0.162 | 1.942 | 0.0522 |
| Hospitalization | 0.8256 | 0.2746 | 3.006 | 0.002647 |
| Intercept | -1.552 | 0.1085 | -14.3 | 2.2e-46 |

*******

**Table 7. From a global perspective. 10% of all medicines are falsified drugs.**

|  | Estimator | Standard error | z | p-value |
| --- | --- | --- | --- | --- |
| Age 30-39 | -0.1083 | 0.1456 | -0.7434 | 0.4573 |
| Age 40-49 | 0.2707 | 0.152 | 1.781 | 0.07492 |
| Cardiovascular diseases | -0.403 | 0.2098 | -1.921 | 0.05472 |
| Hypertension | 0.2168 | 0.1285 | 1.687 | 0.09161 |
| Hospitalization | 0.8763 | 0.2711 | 3.232 | 0.001231 |
| Intercept | -0.6846 | 0.1136 | -6.025 | 1.692e-09 |

***

**Table 8. Community pharmacies are the only place that ensures the secure purchasing of drugs and can guarantee that the drug has not been falsified.**

|  | Estimator | Standard error | z | p-value |
| --- | --- | --- | --- | --- |
| Diabetes | 0.2398 | 0.1423 | 1.685 | 0.09207 |
| Intercept | 0.6213 | 0.07042 | 8.822 | 1.12e-18 |

*******

**Table 9. Purchasing drugs on the Internet is associated with a higher risk of receiving falsified drugs.**

|  | Estimator | Standard error | z | p-value |
| --- | --- | --- | --- | --- |
| Secondary/high school education | 0.302 | 0.2396 | 1.261 | 0.2075 |
| Higher education | -0.2162 | 0.2296 | -0.9416 | 0.3464 |
| Cardiovascular diseases | -0.5334 | 0.2223 | -2.4 | 0.01642 |
| Hospitalization | 1.408 | 0.5292 | 2.66 | 0.007816 |
| Intercept | 1.421 | 0.2086 | 6.815 | 9.441e-12 |

*******

**Table 10. I would be able to distinguish a falsified drug from a non-falsified one.**

|  | Estimator | Standard error | z | p-value |
| --- | --- | --- | --- | --- |
| Cardiovascular diseases | -0.5244 | 0.1941 | -2.702 | 0.00689 |
| Hypertension | -0.2452 | 0.1338 | -1.832 | 0.06692 |
| Diabetes | -0.5253 | 0.1418 | -3.705 | 0.0002116 |
| Financial situation – good | 0.1107 | 0.1674 | 0.6613 | 0.5084 |
| Financial situation – satisfactory | -0.301 | 0.1665 | -1.808 | 0.07062 |
| Financial situation – non- satisfactory | -0.3339 | 0.2608 | -1.281 | 0.2004 |
| Intercept | 0.9461 | 0.1511 | 6.26 | 3.845e-10 |

***

**Table 11. A falsified drug would not hurt, but it also would not help.**

|  | Estimator | Standard error | z | p-value |
| --- | --- | --- | --- | --- |
| Age 30-39 | -0.146 | 0.1397 | -1.045 | 0.296 |
| Age 40-49 | -0.5754 | 0.1508 | -3.816 | 0.0001355 |
| Cardiovascular diseases | -0.2974 | 0.1984 | -1.499 | 0.1339 |
| Hypertension | -0.3453 | 0.1314 | -2.628 | 0.008593 |
| Diabetes | -0.2975 | 0.1422 | -2.092 | 0.03645 |
| Hospitalization | -0.5412 | 0.2879 | -1.88 | 0.06012 |
| Financial situation – good | -0.02825 | 0.1593 | -0.1773 | 0.8593 |
| Financial situation – satisfactory | -0.2721 | 0.1619 | -1.681 | 0.09272 |
| Financial situation – non- satisfactory | -0.7243 | 0.2667 | -2.716 | 0.006603 |
| Intercept | 0.6637 | 0.166 | 3.998 | 6.393e-05 |

*******

**Table 12. Falsified drugs can worsen the health status.**

|  | Estimator | Standard error | z | p-value |
| --- | --- | --- | --- | --- |
| Financial situation – good | 0.09363 | 0.2318 | 0.4039 | 0.6863 |
| Financial situation – satisfactory | 0.002411 | 0.2324 | 0.01037 | 0.9917 |
| Financial situation – non- satisfactory | -1.136 | 0.2965 | -3.831 | 0.0001278 |
| Intercept | 1.865 | 0.1816 | 10.27 | 9.81e-25 |

*******

**Table 13. A falsified drug is as safe as a non-falsified drug.**

|  | Estimator | Standard error | z | p-value |
| --- | --- | --- | --- | --- |
| Secondary/high school education | 0.04422 | 0.2141 | 0.2065 | 0.8364 |
| Higher education | 0.3392 | 0.219 | 1.549 | 0.1214 |
| Cardiovascular diseases | -0.7465 | 0.2003 | -3.728 | 0.000193 |
| Financial situation – good | 0.1478 | 0.2028 | 0.7287 | 0.4662 |
| Financial situation - satisfactory | -0.4218 | 0.195 | -2.163 | 0.03057 |
| Financial situation – non- satisfactory | -0.6457 | 0.2877 | -2.244 | 0.02482 |
| Intercept | 1.347 | 0.2446 | 5.506 | 3.673e-08 |

***

**Table 14. A falsified drug can kill.**

|  | Estimator | Standard error | z | p-value |
| --- | --- | --- | --- | --- |
| Age 30-39 | 0.311 | 0.1511 | 2.058 | 0.03961 |
| Age 40-49 | 0.07683 | 0.1566 | 0.4907 | 0.6236 |
| Cardiovascular diseases | -0.2827 | 0.1945 | -1.454 | 0.146 |
| Intercept | 0.7603 | 0.1074 | 7.078 | 1.467e-12 |

*******

**Table 15. Drugs accelerating weight loss.**

|  | Estimator | Standard error | z | p-value |
| --- | --- | --- | --- | --- |
| Gender – male | -0.3834 | 0.1444 | -2.655 | 0.007936 |
| Intercept | 1.556 | 0.1084 | 14.35 | 1.13e-46 |

*******

**Table 16. Anabolic steroids**

|  | Estimator | Standard error | z | p-value |
| --- | --- | --- | --- | --- |
| Hypertension | -0.1816 | 0.1282 | -1.417 | 0.1565 |
| Diabetes | -0.2181 | 0.1381 | -1.58 | 0.1142 |
| Intercept | 0.6176 | 0.08786 | 7.03 | 2.073e-12 |

*******

**Table 17. Drugs lowering blood pressure**

|  | Estimator | Standard error | z | p-value |
| --- | --- | --- | --- | --- |
| Financial situation - good | 0.1848 | 0.1989 | 0.9293 | 0.3527 |
| Financial situation – satisfactory | 0.2299 | 0.2007 | 1.145 | 0.252 |
| Financial situation – non- satisfactory | 0.7309 | 0.2863 | 2.553 | 0.01068 |
| Intercept | -1.516 | 0.1611 | -9.41 | 4.965e-21 |

***

**Table 18. Medications for diabetes**

|  | Estimator | Standard error | z | p-value |
| --- | --- | --- | --- | --- |
| Diabetes | 0.3512 | 0.1617 | 2.172 | 0.02985 |
| Hospitalization | 0.9383 | 0.276 | 3.4 | 0.0006735 |
| Intercept | -1.617 | 0.09096 | -17.78 | 9.708e-71 |

***

**Table 19. Analgesics**

|  | Estimator | Standard error | z | p-value |
| --- | --- | --- | --- | --- |
| Gender - male | 0.3013 | 0.1169 | 2.577 | 0.009962 |
| Cardiovascular diseases | -0.4997 | 0.1972 | -2.534 | 0.01128 |
| Diabetes | 0.2016 | 0.1341 | 1.504 | 0.1327 |
| Hospitalization | 0.388 | 0.2707 | 1.433 | 0.1518 |
| Intercept | -0.2932 | 0.09322 | -3.145 | 0.00166 |

***

**Table 20. Antibiotics**

|  | Estimator | Standard error | z | p-value |
| --- | --- | --- | --- | --- |
| Hospitalization | 0.7757 | 0.2681 | 2.893 | 0.003812 |
| Intercept | -1.168 | 0.06968 | -16.76 | 4.931e-63 |

***

**Table 21. Antiplatelet and antithrombotic drugs**

|  | Estimator | Standard error | z | p-value |
| --- | --- | --- | --- | --- |
| Hospitalization | 0.9969 | 0.277 | 3.599 | 0.0003192 |
| Intercept | -1.595 | 0.07915 | -20.15 | 2.842e-90 |
